# Effects of Nanotoxicity on Female Reproductivity and Fetal Development in Animal Models

**DOI:** 10.3390/ijms14059319

**Published:** 2013-04-29

**Authors:** Jianling Sun, Qiu Zhang, Zhiping Wang, Bing Yan

**Affiliations:** 1 School of Chemistry and Chemical Engineering, Shandong University, Jinan 250100, China; E-Mails: sjl0000@163.com (J.S.); zhangqiufcn@yahoo.com.cn (Q.Z.); 2 School of Public Health, Shandong University, Jinan 250100, China; E-Mail: zhipingw@sdu.edu.cn

**Keywords:** nanotoxicity, reproductivity, development, animal model, zebrafish

## Abstract

The extensive application of nanomaterials in industry, medicine and consumer products has raised concerns about their potential toxicity. The female population is particularly vulnerable and deserves special attention because toxicity in this group may impact both female reproductivity and fetal development. Mouse and zebrafish models each have their own unique features and studies using these models to examine the potential toxicity of various nanoparticles are compared and summarized in this review. Several nanoparticles exhibit detrimental effects on female reproductivity as well as fetal development, and these adverse effects are related to nanoparticle composition, surface modification, dose, exposure route and animal species. Limited studies on the mechanisms of nanotoxicity are also documented and reviewed herein.

## 1. Introduction

Nanoparticles have unique thermal, mechanical, magnetic and optical properties that allow for their widespread application in biomedicine and many industrial sectors [1–3]. More than 1310 marketed consumer products are based on nanomaterials [4], and this number is rapidly increasing. Nanoparticles cause pulmonary injury [5], hepatotoxicity [6,7], immunotoxicity [8], neurotoxicity [9], renal toxicity [10] and reversible testis damage [11] in animals. Recently, severe pulmonary fibrosis caused by polymer nanoparticles in seven young female workers provided new evidence for nanotoxicity in humans [12]. Consequently, the increasing public and occupational exposure to nanomaterials is a call for concern with respect to nanotoxicity.

Normal female reproduction and fetal development are essential for the perpetuation of the species. However, the female reproductive system is considerably more fragile than other systems, as described below. First, compared with the reproductive male gametes, female gametes are rather limited. During a woman’s lifetime, only about 400 follicles sequentially mature and ovulate [13]. Second, the female reproductive organs, such as the ovary and uterus, exhibit periodic growth and regression, which is strictly regulated by hormones. Its dynamic activity and rigorous hormonal control make this system more sensitive to foreign bodies and physiological stress compared to other physiological processes [14,15]. Third, the disturbance of female reproduction inevitably leads to abnormal fetal development. Many environmental chemicals have already demonstrated detrimental effects on the female reproductive system and embryonic development [15,16].

The emergence of nanoparticles has added a new threat to the vulnerable female population. The toxicity of nanoparticles to female reproductive and developmental health has been studied in various models [17–19]. In this review, we focus on nanotoxicity studies that use mouse and zebrafish models. The genomic similarity between mice and humans is the primary reason for the wide use of mice in the life sciences; the mouse as a mammalian model provides analogous experimental conditions and comparable results to humans, albeit with certain limitations. For example, animals are expensive, the study cycle is rather long, the study throughput is low and several animal protection organizations are active around the world. Furthermore, the investigation of early developmental effects is challenging in mice because the process occurs *in utero* and is thus not easily detectable. In contrast, studies in zebrafish are fast, less expensive and can be modelled using a high-throughput format. Embryonic development in zebrafish can be studied *in vitro*, which allows for testing at all stages [20]. Therefore, the zebrafish has become the model of choice for molecular mechanism studies in embryonic development and one of the most valued models in developmental biology. In the following review, we summarise the main findings of these studies.

## 2. Effects of Nanotoxicity on Female Reproductivity and Development in Murine Models

The mouse is a commonly used animal model for toxicological evaluation. Its genomic similarity to humans as well as its short generation time and large litter size in mammals are the key elements for its extensive use. The availability of multiple species and knockout mice satisfies the needs of many specific studies.

### 2.1. Toxicity to the Female Reproductive System

**Figure 1:**
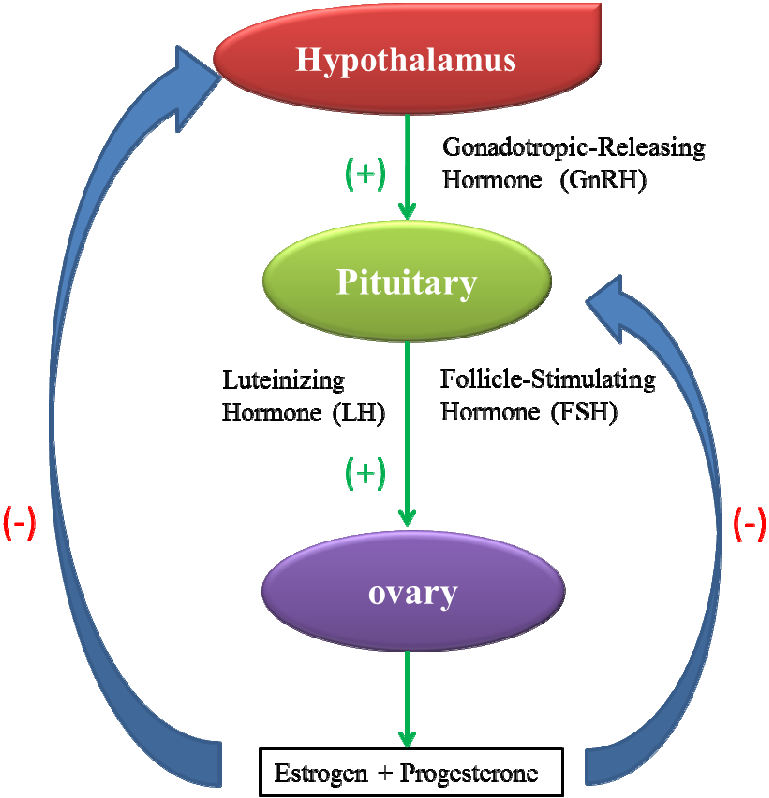
The hypothalamic-pituitary-ovarian axis of the female reproductive system. (+) represents positive feedback, and (−) represents negative feedback.

The mammalian female reproductive system is composed of the hypothalamic-pituitary-ovarian axis and reproductive organs, including the oviducts, uterus, vagina and external genitalia. The normal operation of the reproductive system depends on the precise positive and negative feedback between various components of the axis (Figure 1) [21]. The interference of xenobiotics with the female reproductive system may impair normal gonadal processes, such as oogenesis, ovulation, hormone production by granulosa cells and the structure or function of the accessory reproductive structures [22].

In an experiment conducted in non-pregnant female mice, long-term exposure to TiO_2_ nanoparticles (5–6 nm, intragastric administration) at a concentration of 10 mg/kg was found to cause ovarian dysfunction and alterations in functional gene expression levels. The hormone-related gene Cyp17a1 was up-regulated, indicating the increased biosynthesis of estradiol. Additionally, three genes regulating apoptosis were down-regulated, while eighteen genes such as bmf were up-regulated. Changes in the expression of genes regulating immune and inflammatory responses, oxidative stress, ion transport, cell proliferation, transcription and oxidoreductase activity of the ovary were also observed. TiO_2_ nanoparticles were detected in the ovarian cells of these mice, and the resultant cellular damage led to an imbalance in sex hormones and decreased fertility [23]. In another study [24], the daily inhalation of CdO nanoparticles (230 μg/m^3^) increased the uterine weight and altered the placental weight of pregnant CD-1 mice. Furthermore, reduced levels of 17β-estradiol and altered expression levels of estrogen receptor α and β (ERα and ERβ) in the uterus eventually led to decreased implantation. Cd ions that released from the CdO nanoparticles may act as an endocrine disruptor to prevent implantation and perturb the implanted blastocysts. However, the mechanism of action has still not been verified. The intravenous injection of SiO_2_ and TiO_2_ nanoparticles at a dose of 0.8 mg/mouse in pregnant mice was shown to result in a decreased uterine weight and an increased fetal reabsorption rate [25]. These studies demonstrate that nanoparticles may adversely impact the female reproductive system and fertility, as has been shown for other toxic chemicals [26].

### 2.2. Transplacental Ability of Nanoparticles

**Figure 2:**
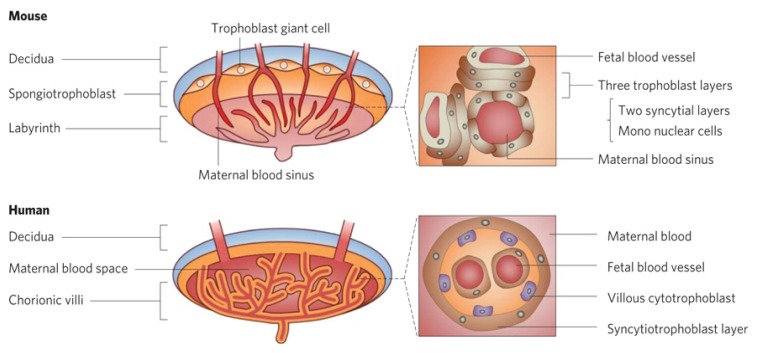
Pathological examination of the mouse and human placenta (reproduced with permission from [25]^©^2011, Nature Publishing Group).

The placenta is a hormonally regulated organ that is responsible for maternal-fetal exchange and is essential for the maintenance of gestation and embryonic growth (Figure 2) [25]. However, the placenta is not an effective barrier, as environmental pollutants and drugs are known causes of birth defects [27,28]. Many nanoparticles, such as Au nanoparticles [29], TiO_2_ nanoparticles [30], SiO_2_ nanoparticles [25,31], quantum dots (QDs) [32] and carbon nanoparticles [33], can also penetrate the placental barrier.

Smaller particles seem to have a stronger transplacental ability than larger particles with an identical chemical composition. When SiO_2_ nanoparticles (70, 300 and 3000 nm) were intravenously injected into pregnant BALB/c mice at a concentration of 0.8 mg/mouse, nanoparticles of all sizes were detected in the liver, while only nanoparticles with a diameter of 70 nm were detected in the placental trophoblasts, the fetal liver and the fetal brain [25]. Similarly, when Au nanoparticles of two different sizes were injected into pregnant rats, the smaller nanoparticles (1.4 nm) were detected in the placenta rather than the larger nanoparticles (18 nm) [29].

In addition to their size, surface modification also regulates the biodistribution of nanoparticles. The transplacental ability of CdTe/CdS QDs was reduced following modification with polyethylene glycol (PEG) or capping with an inorganic silica shell [32]. Similarly, the phenomenon that surface modification regulates the uptake of nanoparticles was also observed when tested with mouse embryos *in vitro* [34]. In this study, amine- and carboxyl-modified polystyrene beads of different sizes (200 nm for the former, and 20, 100, 500 nm for the latter) were injected into the extra-embryonic tissue of cultured embryos *in vitro*. The results showed that carboxylic polystyrene beads with a diameter greater than 100 nm were located solely in the extra-embryonic tissue, while 200 nm amine-modified beads crossed into the embryos.

Therefore, the transplacental ability of nanoparticles is dependent on size and surface modification [33–36]. A human placental perfusion model study confirmed that nanoparticles have the ability to cross the placenta by means of endocytosis [37]. Placental damage caused by nanoparticles may potentially lead to the deformity or developmental retardation of the fetus.

### 2.3. Effects of Nanoparticles on Fetal Developmental

Although nanoparticles may cause damage to embryos as a result of their transplacental ability [38], they may also affect the offspring through altered signaling pathways. Maternal exposure to carbon black nanoparticles (100 μg/mouse) by intranasal instillation of ICR mice was found to induce the overexpression of renal type VIII collagen in the offspring [39]. Inhalation of TiO_2_ nanoparticles (1 h/day, 42 mg/m^3^) by pregnant C57BL/6 mice from gestational day (GD) 8 to18 resulted in the abnormal expression of genes of the retinoic acid signaling pathway in the livers of newborn female mice [40]. Nanoparticles may also cause altered organogenesis and morphology as well as defects in the reproductive and nervous systems of the offspring (Table 1). These effects are discussed in the table below.

#### 2.3.1. Toxicity on Fetal Organogenesis and Morphology

**Figure 3:**
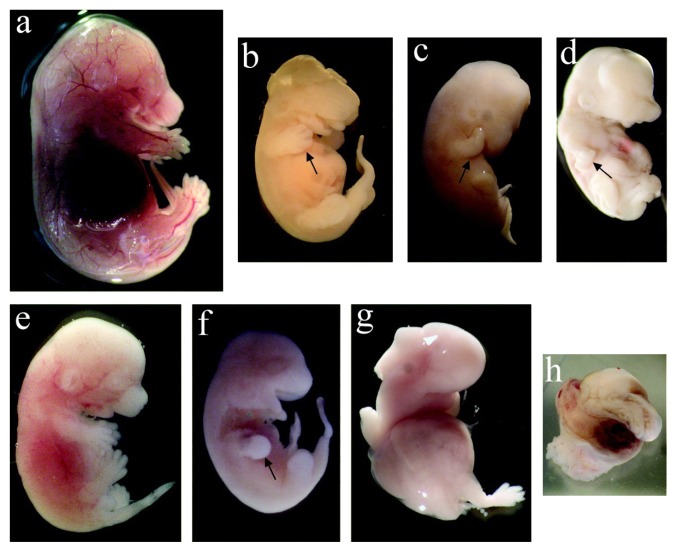
Exposure of pregnant mice to SWCNTs causes malformed fetuses. (**a**) Normal fetus; (**b**–**h**) Malformed fetuses from SWCNT groups (reproduced with permission from [52]^©^2011, American Chemical Society).

Exposure to nanoparticles during the gestational period affects fetal organogenesis and morphology [17]. With intravenous administration, both pristine and oxidized single-walled carbon nanotubes (SWCNTs) with concentrations varying from 10 ng to 30 μg/mouse was observed to induce morphological abnormalities in the fetuses of pregnant CD-1 mice. The fetuses exhibited deformities in the abdominal wall or head, retarded development of the limbs and snout, swollen abdomens with abnormal torsion of the trunks (Figure 3). Furthermore, oxidized SWCNTs caused more abnormalities in the fetuses than did pristine SWCNTs [52]. The intragastric administration of hydroxyl-modified SWCNTs (10 mg/kg) to pregnant CD-1 mice was demonstrated to cause increased skeletal defects, such as forked cervical vertebrae, reduced ossification of sternebra and phalanges and morphological abnormalities [41]. However, toxic effects were not observed when multi-walled carbon nanotubes (MWCNTs) were administered by gavage to pregnant Sprague-Dawley rats. The offspring of the tested group showed no differences in morphological, visceral or skeletal malformations compared with the control group. The no-observed-adverse-effect level for embryonic-fetal development is considered to be 1000 mg/kg/day [53].

To mimic the exposure of females to nanoparticles during their entire pregnancy, a study was conducted using platinum nanoparticles from pre-gestation to post-delivery. Platinum nanoparticles (0.25, 0.5 and 1 mg/kg) were orally administered to ICR mice from 14 days before mating to 4 days after delivery. Decreased growth and increased mortality of pups during the lactation period were observed, although no deformity was observed in any of the pups [54].

#### 2.3.2. Toxicity to the Fetal Reproductive Function

Diesel exhaust (DE) is generated from the combustion of diesel fuel and is comprised of fine and ultrafine particles. DE has been shown to disrupt the reproductive development of offspring [55,56]. Pre- and postnatal exposure of ICR mice to DE at a dose of 0.17 mg/m^3^caused a reduced daily sperm production (DSP) and deformed the sertoli and spermatozoa cells in the offspring [57].

Because DE contains various compounds and particles, its reactive components were evaluated. Nanoparticle-rich DE (148.86 μg/m^3^, 5 h/day) and filtered DE (3.10 μg/m^3^, 5 h/day) were administered to pregnant F344 rats from GD 1 to GD 19. In both groups, the organ indices of the seminal vesicle and prostate and the concentrations of various hormones, such as testosterone, progesterone, corticosterone, LH and FSH, in the serum of the male offspring decreased. Testicular histology and real-time reverse transcription polymerase chain reaction analysis showed a loss of germ cells in the seminiferous tubules and altered expression of steroidogenic acute regulatory protein, 17β-hydroxysteroid dehydrogenase and follicle-stimulating hormone receptor mRNA [45]. The alterations observed in both groups were similar, indicating that the gaseous phase of the exhaust, rather than the particles, played a major role in disrupting the reproductive function. The hormone-like activity of the compounds adsorbed to the particles disturbed the hormone levels and thereby restricted growth [58]. Thus, the particles only served as carriers of the hazardous compounds.

The exposure of pregnant C57BL/6BomTac mice to DE (20 mg/m^3^, 1 h/day) from GD 7 to GD 19 caused a reduced DSP in the adult offspring. However, the testis weight, concentrations of testosterone and estradiol, gene expression of hormone-related receptors were not altered compared with the controls. These results indicate that *in utero* exposure to DE may not affect the endocrine activity in adult offspring [44], although another report suggested an endocrine-like activity for DE [45].

#### 2.3.3. Effects on Fetal Neurodevelopment

Traffic-related air pollution may cause adverse effects on neurodevelopment in children [59]. Prenatal exposure of pregnant mice to nanoparticles caused neurological disorders in their offspring [50,60]. In an inhalation study, prenatal exposure to DE (0.3, 1 and 3 mg/m^3^) resulted in various types of damage, including caspase-3-positive cells in the cerebral cortex and hippocampus and crescent-shaped spaces in some cells. Furthermore, the granular epithelial cells and scavenger cells that constitute the blood brain barrier (BBB) underwent apoptosis [49]. Maternal exposure to TiO_2_ (0.1 mg/mouse) by subcutaneous injection also resulted in the apoptosis of endothelial cells, capillary stenosis and degenerative changes in the neighboring parenchyma [30,60]. The nanoparticle-induced reduction of dopamine (DA) turnover in the nucleus accumbens and striatum induced a decrease in spontaneous motor activity, thereby emphasizing the adverse effects of TiO_2_ nanoparticles on the central dopaminergic system [48]. Analysis of the gene expression in the brain of the offspring indicated that the alterations are related to inflammation, oxidative stress and neurotransmitters [47].

Several methods have been used to evaluate offspring behavior after the treatment of pregnant mice with nanoparticles. The open field test is used to test locomotor activity, the Morris water maze is used to test learning and memory and the acoustic startle test is used to test sensorimotor function. Using the open field test, prenatal exposure of ICR mice to DE (1.0 mg/m^3^, 8 h/day) from GD 2 to GD 17 decreased the spontaneous motor activity in the male offspring [48]. The Morris water maze test showed that the cognitive ability of female offspring was enhanced after maternal DE (19 mg/m^3^, 1 h/day) exposure from GD 9 to GD 19 [50]. Intratracheal implantation of carbon black (268 μg/mouse) in pregnant mice led to no effect on acoustic startle of their offspring [51].

#### 2.3.4. Mechanistic Studies

Reactive oxygen species (ROS) are generated from molecular oxygen through multiple perturbations [61]. Excessive ROS overwhelms the cellular antioxidant capacity and leads to cellular injuries and malignant diseases [62,63]. According to the hierarchical oxidative stress model [64], an intermediate amount of ROS induces inflammatory responses through the MAPK and NF-κB signaling cascades [65,66]. Nanoparticle-generated ROS has been identified as a source of female reproductive and developmental toxicity [67–69]. The increased level of ROS reacts with biomacromolecules (DNA, protein, lipids), disturbs intracellular homeostasis, triggers apoptosis [70] and eventually leads to maternal and embryonic toxicity.

Maternal exposure to nanoparticles likely causes fetal dysfunction in two ways: (1) nanoparticles are transferred to the fetus through blood circulation, where they result in the production of ROS; and (2) nanoparticles generate ROS in the mother, and the resulting inflammatory cytokines affect the fetus [69]. Oxidative stress produced in the fetus is the dominating factor of nanoparticle-mediated teratogenesis. Because ROS is not stable enough to travel through the cell [64], ROS in the mother may indirectly contribute to embryonic dysfunction [38,51,54,71]. The balance between oxidation, antioxidation and damage repair determines ROS-mediated risk. Except for the indirect effects from ROS and inflammation, nanoparticles may also cause direct DNA damage following nuclear translocation [72,73].

### *2.4. In Vitro* Models of Murine Origin

Blastocysts and granulosa cells have been used to evaluate the reproductive toxicity of nanoparticles *in vitro*. Ag nanoparticles (13 nm) and CdSe-core QDs (3.5 nm) were shown to inhibit cell proliferation and increase apoptosis in blastocysts [74,75]. Compared to the controls, treatment with Ag nanoparticles and QDs affected embryonic development post-implantation. Blastocysts pretreated with either the Ag nanoparticles (50 μM) or QDs (500 nmol/L) induced a high resorption rate of post-implantation embryos and a decrease in fetal weight. Granulosa cells, which are responsible for follicular growth, are the primary functional cells in the ovary. It was observed that Au nanoparticles (2.85 × 10^10^ particles/mL medium) can enter the ovarian granulosa cells and inhibit the synthesis of estradiol [76]. All of these *in vitro* studies further confirm the potential toxicity of nanoparticles on reproduction and early embryonic development.

## 3. Nanotoxicity to Female Reproductivity and Embryonic Development in Zebrafish

Zebrafish is a well-known model for the evaluation of reproduction and development and can be used for studying the biodistribution and potential toxicity of nanoparticles. Zebrafish possess several advantages as a model for studying vertebrate development. First, their genome shares significant homology with the human genome [77]; thus, it is possible to study various physiological functions and biological processes, such as angiogenesis, malformation (including pericardial oedema and bent notochords) and oxidative stress caused by foreign substances. Second, theirs small size and high fecundity make zebrafish a cost-effective model for research [78]. Approximately 200–300 eggs can be produced from a single mating every 5–7 days, providing a sufficient number of organisms and a reduced experimental time compared to mice. In mice, the gestational period is approximately 20 days, and their litter size is far less than zebrafish. Third, transparent embryos and a rapid embryonic development facilitate the observation of morphogenetic changes and organogenesis in real time [79,80]. Fourth, various mutant phenotypes of zebrafish are available to link gene function with the corresponding pathophysiology [20,81]. These advantages make zebrafish a cheaper and time-saving alternative to rodent models.

### 3.1. Translocation of Nanoparticles to the Zebrafish Embryo

**Figure 4:**
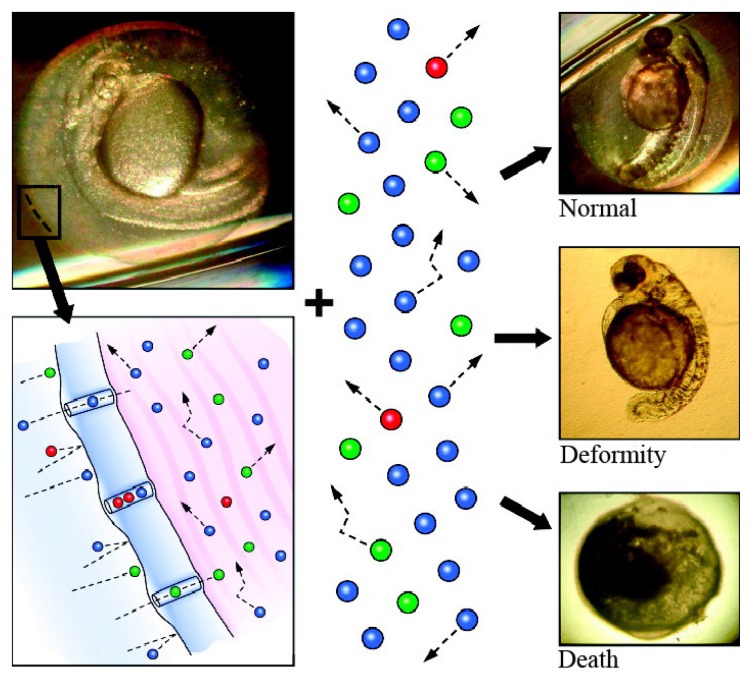
Single Ag nanoparticles diffuse through the chorion pore canals and are observed in normal, deformed and dead zebrafish (reproduced with permission from [84]^©^2007, American Chemical Society).

The chorion is a three-layered acellular envelope surrounding the embryo [82] that protects the embryo from foreign interference during development before hatching [83]. The chorion pore canals are critical for the transport of nutrients and oxygen from the aquatic environment to the embryo and for the excretion of waste in the opposite direction. Scanning-electron microscopy has shown that the pore size is 0.5–0.7 μm in diameter; thus, small nanoparticles may enter the embryo through these canals (Figure 4) [84]. It was observed that single Ag nanoparticles (5–46 nm and 11.3 ± 2.3 nm) enter the chorionic space of the embryo by Brownian diffusion [84,85], while SWCNT agglomerates are too large to cross the canals [86]. The SWCNTs were found to aggregate in the culture medium and formed agglomerates ranging in size from several hundred nanometers to several micrometers at the outer layer of the chorion. Similar results were observed when fluorescent SiO_2_ nanoparticles (~60 and ~200 nm in diameter) were exposed to zebrafish embryos; the nanoparticles adhered to the surface of the chorion but did not enter the embryo [87].

### 3.2. Toxicity of Nanoparticles in Zebrafish Embryos

Zebrafish embryos have been used to evaluate developmental toxicity and biocompatibility in their rapid growth phase. Following treatment with nanoparticles, the embryos are placed in microtiter plates, and their survival states and phenotypic changes are examined. As a predictive model, zebrafish have been used to assess the nanotoxicity of metal and metal oxide nanoparticles, QDs and carbon nanomaterials.

#### 3.2.1. Metal Nanoparticles

Both Ag and platinum nanoparticles were shown to cause concentration-dependent hatching delays, mortality, a drop in heart rate and other abnormalities. Furthermore, exposure to Ag nanoparticles (5–35 nm) caused defective eyes or eyeless phenotypes, pericardial oedema, pericardial effusion and circulatory defects starting at a concentration of 25 mg/mL [88]. The critical concentration of Ag nanoparticles (5–46 nm) that resulted in embryonic abnormalities and death was determined to be 1.9 nM [84]. Surface modifications, such as cysteine, decreased the toxicity of Ag nanoparticles in zebrafish [89]. It was also observed that Ag nanoparticles are more toxic than Au nanoparticles in zebrafish [88]. The teratogenicity and lethality of Au nanoparticles vary with the surface coating [90], and they show more biocompatibility than Ag nanoparticles [91,92].

The microinjection of TiO_2_ nanoparticles (<25 nm, 8.5 ng/g) into zebrafish embryos was observed to down-regulate the expression of genes that regulate the circadian rhythm, kinase-related activities, the immune response and vesicular transport [93]. However, embryonic incubation with a suspension of TiO_2_ nanoparticles (≤20 nm) displayed no toxic effects up to a concentration of 500 μg/mL [94]. In addition to the nanotoxicity of TiO_2_ nanoparticles, photocatalysis of TiO_2_ under illumination produces ROS, offering another key factor for toxicity. When zebrafish embryos were incubated with TiO_2_ nanoparticles (23.3 nm), the half lethal concentrations (LC_50_) of the TiO_2_ nanoparticles with/without illumination were determined to be 300 μg/mL and >1000 μg/mL, respectively, at 120 h post-fertilization (hpf) [95].

ZnO nanoparticles (20 nm) exhibit toxicity in zebrafish embryos, and the 96 hpf LC_50_ of their suspension was observed to be 1.793 mg/mL [94,96]. QDs have potent toxicity due to their small size and heavy metal content [97–101]. Studies from different laboratories have shown that the exposure of zebrafish to QDs causes a decreased hatch rate, malformation, a slower heart rate and delayed growth.

#### 3.2.2. Carbon Nanomaterials

Carboxyl-functionalized MWCNTs caused mortality and alter gene expression in zebrafish embryos. Incubation with MWCNTs (30–40 nm) at a concentration of 60 μg/mL induced hatching delays and an inflammatory response. Detrimental effects on the cardiovascular system, an increased mortality rate, and apoptosis in treated embryos were also observed [102]. The length and aggregation status of CNTs are the factors that influence their toxicity [86,103].

Fullerene (C_60_) exposure was observed to lead to malformations and mortality in zebrafish embryos and also to induce a concentration-dependent increase in cell death (both necrosis and apoptosis) in the head and trunk. In contrast, C_60_ (OH)_24_ was shown to cause much less cell death (no apoptosis) in the head region [104]. Oxidative stress elicited by C_60_ was identified as a factor for malformation in zebrafish embryos [105,106].

## 4. Conclusions

Nanoparticles are capable of inducing detrimental effects on the reproductive systems of mice and zebrafish as well as their fetal development both *in vivo* and *in vitro*. In mice, maternal exposure to nanoparticles causes their localization in the embryo through transplacental transfer and results in fetal toxicity, such as physical defects, neurotoxicity and reproductive toxicity in the offspring. In zebrafish, incubation or microinjection of a low dose of nanoparticles induces hatching delays, mortality, axis curvatures and various morphological defects. Although the consequences of toxicity in these two models are not directly comparable, the toxicity of nanomaterials in diverse living species further highlights the universality of nanotoxicity in animals. The toxicity of nanoparticles is dose- and size-dependent [34,89]. For some nanoparticles (such as TiO_2_ nanoparticles), the crystal forms and illumination are also important [107]. Oxidative stress induced by nanoparticles is considered to be a main factor for female reproductive and developmental toxicity.

Numerous studies have confirmed the toxicity of nanoparticles in different species, and utilizing approaches to make nanomaterials safer will ensure their future use. The biodistribution of many nanoparticles is size-dependent; nanoparticles with a small particle size are better able to make contact with their functional units to exert their effects. However, their size may not necessarily determine their toxicity [108]. Therefore, a compromise between the size of the nanoparticles, their function (as in sewage disposal or medical applications) and their side effects could be one way to alleviate their toxicity. In addition, their shape and aggregation potential could also regulate the toxicity of nanoparticles. Plate-shaped Ag nanoparticles exhibit more severe toxicity on zebrafish embryos than nanospheres and nanowires due to the high levels of crystal defects on the surface of the plate [89]. The 10% and median lethal concentration (LD_10_ and LD_50_) of dendritic clusters of 60 nm nickel nanoparticles in zebrafish embryos were found to be much lower than well-dispersed 60 nm nanospheres, which showed a higher level of toxicity when aggregated [109]. Furthermore, surface modification is the most common method used to obtain nanoparticles with a low toxicity [90]. The toxicity induced by C_60_, QDs, polyamidoamine dendrimers and lead sulfide nanoparticles can also be regulated by functionalization. A change in the chemical components during manufacture could be another route to reduce toxicity; for instance, ZnO nanoparticles doped with iron showed reduced detrimental effects in mice and zebrafish.

Investigations on the effects of nanotoxicity on female reproductivity and fetal development have several issues. First, some of the reported results contradict each other, possibly due to differences in the quality of the nanoparticles used, variable experimental protocols and the different animal species selected. Unified standards, protocols and coordinated efforts are greatly needed. Second, the investigations to date have been limited to one generation. Multi-generation investigations should be launched in the near future to evaluate the long-term influence of nanoparticles. Third, because of the limited data on the reproductive and developmental toxicity of nanoparticles in humans, the use of animal models may help to speculate their effects. However, we should recognise the structural distinctions between experimental models and humans. For example, the duration of gestation in the mouse is only 20 days compared with 10 months in humans. Moreover, the reproductive structures and endocrine functions are different between mice and humans [110,111]. Therefore, conclusions derived from animal models should not be extrapolated to humans without considering their physiological differences. In short, research in this field is still quite preliminary. With more sophisticated studies, a better understanding of female reproductive and developmental toxicity will emerge in the near future.

## Figures and Tables

**Table 1:** Fetal toxicity of nanoparticles in murine models.

Nanoparticles/characteristics	Mouse strain	Exposure	Dose	Duration	Toxicity in offspring	Ref.
SWCNT, 1–2 nm in diameter, 5–30 μm in length	CD-1	Oral gavage	5, 10 or 100 mg/kg	GD 9	Skeletal abnormalities and external defects	[41]
TiO_2_, rutile, 21 nm, coated with polyalcohol	C57BL/6BomTac	Inhalation	42 mg/m^3^	GD 8–18, 1 h/day	Avoidance of the central zone in the open field test; enhanced prepulse inhibition in female offspring	[42]
TiO_2_, anatase, 25–70 nm, surface area of 20–25 m^2^/g	Slc:ICR	Subcutaneous injection	100 μg/mouse	3, 7, 10 and 14 days post-coitus	Decreased daily sperm production and sperm motility; disorganised and disrupted seminiferous tubules; apoptosis in the olfactory bulb	[30]
Carbon nanoparticles, 14 nm	ICR	Intratracheal injection	200 μg/mouse	GD 7 and14	Decreased daily sperm production	[43]
Diesel exhaust	C57BL/6BomTac	Inhalation	20 mg/m^3^, 1 × 10^6^ particles/cm^3^	GD 7–19, 1 h/day	Decreased daily sperm production	[44]
Nanoparticle-rich diesel exhaust, filtered diesel exhaust	F344/DuCrlCrli	Inhalation	Nanoparticle-rich DE: 168.84 μg/m^3^, 1.36 × 10^6^ particles/cm^3^; filtered-DE: 3.1 μg/m^3^, 2.66 particles/cm^3^	GD 1–19	Decreased seminal vesicle and prostate organ index; decreased testosterone, progesterone, corticosterone and FSH levels; altered steroidogenic acute regulatory protein, 17β-hydroxysteroid dehydrogenase and follicle-stimulating hormone receptor mRNA	[45]
TiO_2_, anatase, 25–70 nm, surface area of 20–25 m^2^/g	ICR	Subcutaneous injection	0.1 mL, 1 mg/mL	GD 6, 9, 12, 15 and 18	Increased DA and metabolites in the prefrontal cortex and neostriatum	[46]
TiO2, anatase, 25–70 nm, surface area of 20–25 m2/g	ICR	Subcutaneous injection	100 μL, 1 mg/mL	GD 6, 9, 12 and 15	Altered gene expression associated with apoptosis, oxidative stress and neurotransmitters in the brain	[47]
Diesel exhaust	ICR	Inhalation	1.0 mg/m^3^	GD2–17, 8 h/day, 5 days per week	Reduced locomotion; decreased DA turnover in the striatum and nucleus accumbens	[48]
Diesel exhaust	ICR	Inhalation	0.3, 1 and 3.0 particles/m^3^	2 to 16 days post-coitus	Apoptosis in brain tissue	[49]
Diesel exhaust, 240 nm	C57BL/6 BomTac	Inhalation	19 mg/m^3^, 1 × 10^6^ particles/cm^3^	GD 9–19, 1 h/day	Increased activity in female DE offspring	[50]
Carbon black, average zeta potential of 140 nm, hydrodynamic size of 50–60 nm	C57BL/6BomTac	Intratracheal instillation	11, 54 and 268 μg/animal	GD 7, 10, 15 and 18	Altered habituation pattern in female offspring	[51]

## References

[1] Zhang L., Gu F., Chan J., Wang A., Langer R., Farokhzad O. (2007). Nanoparticles in medicine: Therapeutic applications and developments. Clin. Pharmacol. Ther.

[2] Lee J.H., Huh Y.M., Jun Y., Seo J., Jang J., Song H.T., Kim S., Cho E.J., Yoon H.G., Suh J.S. (2006). Artificially engineered magnetic nanoparticles for ultra-sensitive molecular imaging. Nat. Med.

[3] Das S.K., Das A.R., Guha A.K. (2009). Gold nanoparticles: Microbial synthesis and application in water hygiene management. Langmuir.

[4] An inventory of nanotechnology-based consumer products currently on the market.

[5] Chou C.C., Hsiao H.Y., Hong Q.S., Chen C.H., Peng Y.W., Chen H.W., Yang P.C. (2008). Single-walled carbon nanotubes can induce pulmonary injury in mouse model. Nano Lett.

[6] Derfus A.M., Chan W.C.W., Bhatia S.N. (2004). Probing the cytotoxicity of semiconductor quantum dots. Nano Lett.

[7] Bartneck M., Ritz T., Keul H.A., Wambach M., Bornemann J., Gbureck U., Ehling J., Lammers T., Heymann F., Gassler N. (2012). Peptide-functionalized gold nanorods increase liver injury in hepatitis. ACS Nano.

[8] Schipper M.L., Nakayama-Ratchford N., Davis C.R., Kam N.W.S., Chu P., Liu Z., Sun X., Dai H., Gambhir S.S. (2008). A pilot toxicology study of single-walled carbon nanotubes in a small sample of mice. Nature Nanotech.

[9] Wu J., Wang C., Sun J., Xue Y. (2011). Neurotoxicity of silica nanoparticles: Brain localization and dopaminergic neurons damage pathways. ACS nano.

[10] Lin P., Chen J.W., Chang L.W., Wu J.P., Redding L., Chang H., Yeh T.K., Yang C.S., Tsai M.H., Wang H.J. (2008). Computational and ultrastructural toxicology of a nanoparticle, Quantum Dot 705, in mice. Environ. Sci. Technol.

[11] Bai Y., Zhang Y., Zhang J., Mu Q., Zhang W., Butch E.R., Snyder S.E., Yan B. (2010). Repeated administrations of carbon nanotubes in male mice cause reversible testis damage without affecting fertility. Nature Nanotech.

[12] Song Y., Li X., Du X. (2009). Exposure to nanoparticles is related to pleural effusion, pulmonary fibrosis and granuloma. Eur. Respir. J.

[13] Hillier S. (1994). Current concepts of the roles of follicle stimulating hormone and luteinizing hormone in folliculogenesis. Hum. Reprod.

[14] Warren M., Perlroth N. (2001). The effects of intense exercise on the female reproductive system. J. Endocrinol.

[15] Armenti A.E., Zama A.M., Passantino L., Uzumcu M. (2008). Developmental methoxychlor exposure affects multiple reproductive parameters and ovarian folliculogenesis and gene expression in adult rats. Toxicol. Appl. Pharm.

[16] Anway M.D., Cupp A.S., Uzumcu M., Skinner M.K. (2005). Epigenetic transgenerational actions of endocrine disruptors and male fertility. Science.

[17] Tsuchiya T., Oguri I., Yamakoshi Y.N., Miyata N. (1996). Novel harmful effects of [60]fullerene on mouse embryos *in vitro* and *in vivo*. FEBS Lett.

[18] Wang J., Zhu X., Zhang X., Zhao Z., Liu H., George R., Wilson-Rawls J., Chang Y., Chen Y. (2011). Disruption of zebrafish (Danio rerio) reproduction upon chronic exposure to TiO_2_ nanoparticles. Chemosphere.

[19] Shoults-Wilson W.A., Reinsch B.C., Tsyusko O.V., Bertsch P.M., Lowry G.V., Unrine J.M. (2011). Effect of silver nanoparticle surface coating on bioaccumulation and reproductive toxicity in earthworms (Eisenia fetida). Nanotoxicology.

[20] Dooley K., Zon L.I. (2000). Zebrafish: A model system for the study of human disease. Curr. Opin. Genet. Dev.

[21] Apter D. (1997). Development of the hypothalamic-pituitary-ovarian axis. Ann. N. Y. Acad. Sci.

[22] Mattison D., Plowchalk D., Meadows M., Al-Juburi A., Gandy J., Malek A. (1990). Reproductive toxicity: Male and female reproductive systems as targets for chemical injury. Med. Clin. N. Am.

[23] Gao G., Ze Y., Li B., Zhao X., Zhang T., Sheng L., Hu R., Gui S., Sang X., Sun Q. (2012). Ovarian dysfunction and gene-expressed characteristics of female mice caused by long-term exposure to titanium dioxide nanoparticles. J. Hazard. Mater.

[24] Blum J.L., Xiong J.Q., Hoffman C., Zelikoff J.T. (2012). Cadmium associated with inhaled cadmium oxide nanoparticles impacts fetal and neonatal development and growth. Toxicol. Sci.

[25] Yamashita K., Yoshioka Y., Higashisaka K., Mimura K., Morishita Y., Nozaki M., Yoshida T., Ogura T., Nabeshi H., Nagano K. (2011). Silica and titanium dioxide nanoparticles cause pregnancy complications in mice. Nature Nanotech.

[26] Martino-Andrade A.J., Chahoud I. (2009). Reproductive toxicity of phthalate esters. Mol. Nutr. Food Res.

[27] Olivero O.A., Yuspa S.H., Poirier M.C., Anderson L.M., Jones A.B., Wang C., Diwan B.A., Haines D.C., Logsdon D., Harbaugh S.W. (1997). Transplacental effects of 3′-azido-2′, 3′-dideoxythymidine (AZT): Tumorigenicity in mice and genotoxicity in mice and monkeys. J. Natl. Cancer Inst.

[28] Perera F.P., Rauh V., Tsai W.Y., Kinney P., Camann D., Barr D., Bernert T., Garfinkel R., Tu Y.H., Diaz D. (2003). Effects of transplacental exposure to environmental pollutants on birth outcomes in a multiethnic population. Environ. Health Persp.

[29] Semmler-Behnke M., Fertsch S., Schmid G., Wenk A., Kreyling W.G. Uptake of 1.4 nm versus 18 nm gold nanoparticles in secondary target organs is size dependent in control and pregnant rats after intratracheal or intravenous application.

[30] Takeda K., Suzuki K., Ishihara A., Kubo-Irie M., Fujimoto R., Tabata M., Oshio S., Nihei Y., Ihara T., Sugamata M. (2009). Nanoparticles transferred from pregnant mice to their offspring can damage the genital and cranial nerve systems. J. Health Sci.

[31] Refuerzo J.S., Godin B., Bishop K., Srinivasan S., Shah S.K., Amra S., Ramin S.M., Ferrari M. (2011). Size of the nanovectors determines the transplacental passage in pregnancy: Study in rats. Am. J. Obstet. Gynecol.

[32] Chu M., Wu Q., Yang H., Yuan R., Hou S., Yang Y., Zou Y., Xu S., Xu K., Ji A. (2010). Transfer of quantum dots from pregnant mice to pups across the placental barrier. Small.

[33] Sumner S.C.J., Fennell T.R., Snyder R.W., Taylor G.F., Lewin A.H. (2010). Distribution of carbon-14 labeled C60 ([14C] C60) in the pregnant and in the lactating dam and the effect of C60 exposure on the biochemical profile of urine. J. Appl. Toxicol.

[34] Tian F., Razansky D., Estrada G.G., Semmler-Behnke M., Beyerle A., Kreyling W., Ntziachristos V., Stoeger T. (2009). Surface modification and size dependence in particle translocation during early embryonic development. Inhal. Toxicol.

[35] Morgan K. (2005). Development of a preliminary framework for informing the risk analysis and risk management of nanoparticles. Risk Anal.

[36] Zhang L., Fischer W., Pippel E., Hause G., Brandsch M., Knez M. (2011). Receptor-mediated cellular uptake of nanoparticles: A switchable delivery system. Small.

[37] Wick P., Malek A., Manser P., Meili D., Maeder-Althaus X., Diener L., Diener P.-A., Zisch A., Krug H.F., von Mandach U. (2009). Barrier capacity of human placenta for nanosized materials. Environ. Health Persp.

[38] Fujimoto A., Tsukue N., Watanabe M., Sugawara I., Yanagisawa R., Takano H., Yoshida S., Takeda K. (2005). Diesel exhaust affects immunological action in the placentas of mice. Environ. Toxicol.

[39] Umezawa M., Kudo S., Yanagita S., Shinkai Y., Niki R., Oyabu T., Takeda K., Ihara T., Sugamata M. (2011). Maternal exposure to carbon black nanoparticle increases collagen type VIII expression in the kidney of offspring. J. Toxicol. Sci.

[40] Jackson P., Halappanavar S., Hougaard K.S., Williams A., Madsen A.M., Lamson J.S., Andersen O., Yauk C., Wallin H., Vogel U. (2012). Maternal inhalation of surface-coated nanosized titanium dioxide (UV-Titan) in C57BL/6 mice: Effects in prenatally exposed offspring on hepatic DNA damage and gene expression. Nanotoxicology.

[41] Philbrook N.A., Walker V.K., Afrooz A.R.M.N., Saleh N.B., Winn L.M. (2011). Investigating the effects of functionalized carbon nanotubes on reproduction and development in Drosophila melanogaster and CD-1 mice. Reprod. Toxicol.

[42] Hougaard K.S., Jackson P., Jensen K.A., Sloth J.J., Löschner K., Larsen E.H., Birkedal R.K., Vibenholt A., Boisen A.-M.Z., Wallin H. (2010). Effects of prenatal exposure to surface-coated nanosized titanium dioxide (UV-Titan). A study in mice. Part. Fiber Toxicol..

[43] Yoshida S., Hiyoshi K., Oshio S., Takano H., Takeda K., Ichinose T. (2010). Effects of fetal exposure to carbon nanoparticles on reproductive function in male offspring. Fertil. Steril.

[44] Hemmingsen J.G., Hougaard K.S., Talsness C., Wellejus A., Loft S., Wallin H., Møller P. (2009). Prenatal exposure to diesel exhaust particles and effect on the male reproductive system in mice. Toxicology.

[45] Li C.M., Taneda S., Taya K., Watanabe G., Li X., Fujitani Y., Nakajima T., Suzuki A.K. (2009). Effects of in utero exposure to nanoparticle-rich diesel exhaust on testicular function in immature male rats. Toxicol. Lett.

[46] Takahashi Y., Mizuo K., Shinkai Y., Oshio S., Takeda K. (2010). Prenatal exposure to titanium dioxide nanoparticles increases dopamine levels in the prefrontal cortex and neostriatum of mice. J. Toxicol. Sci.

[47] Shimizu M., Tainaka H., Oba T., Mizuo K., Umezawa M., Takeda K. (2009). Maternal exposure to nanoparticulate titanium dioxide during the prenatal period alters gene expression related to brain development in the mouse. Part. Fiber Toxicol..

[48] Yokota S., Mizuo K., Moriya N., Oshio S., Sugawara I., Takeda K. (2009). Effect of prenatal exposure to diesel exhaust on dopaminergic system in mice. Neurosci. Lett.

[49] Sugamata M., Ihara T., Takano H., Oshio S., Takeda K. (2006). Maternal diesel exhaust exposure damages newborn murine brains. J. Health Sci.

[50] Hougaard K.S., Jensen K.A., Nordly P., Taxvig C., Vogel U., Saber A.T., Wallin H. (2008). Effects of prenatal exposure to diesel exhaust particles on postnatal development, behavior, genotoxicity and inflammation in mice. Part. Fibre Toxicol..

[51] Jackson P., Vogel U., Wallin H., Hougaard K.S. (2011). Prenatal exposure to carbon black (Printex 90): Effects on sexual development and neurofunction. Basic Clin. Pharmacol.

[52] Pietroiusti A., Massimiani M., Fenoglio I., Colonna M., Valentini F., Palleschi G., Camaioni A., Magrini A., Siracusa G., Bergamaschi A. (2011). Low doses of pristine and oxidized single wall carbon nanotubes affect mammalian embryonic development. ACS Nano.

[53] Lim J.-H., Kim S.-H., Shin I.-S., Park N.-H., Moon C., Kang S.-S., Kim S.-H., Park S.-C., Kim J.-C. (2011). Maternal exposure to multi-wall carbon nanotubes does not induce embryo-fetal developmental toxicity in rats. Birth Defects Res. B.

[54] Park E.J., Kim H., Kim Y., Park K. (2010). Effects of platinum nanoparticles on the postnatal development of mouse pups by maternal exposure. Evn. Heal.Toxicol.

[55] Tsukue N., Tsubone H., Suzuki A.K. (2002). Diesel exhaust affects the abnormal delivery in pregnant mice and the growth of their young. Inhal. Toxicol.

[56] Ono N., Oshio S., Niwata Y., Yoshida S., Tsukue N., Sugawara I., Takano H., Takeda K. (2007). Prenatal exposure to diesel exhaust impairs mouse spermatogenesis. Inhal. Toxicol.

[57] Kubo-Irie M., Oshio S., Niwata Y., Ishihara A., Sugawara I., Takeda K. (2011). Pre-and postnatal exposure to low-dose diesel exhaust impairs murine spermatogenesis. Inhal. Toxicol.

[58] Watanabe N. (2005). Decreased number of sperms and Sertoli cells in mature rats exposed to diesel exhaust as fetuses. Toxicol. Lett.

[59] Freire C., Ramos R., Puertas R., Lopez-Espinosa M.J., Julvez J., Aguilera I., Cruz F., Fernandez M.F., Sunyer J., Olea N. (2010). Association of traffic-related air pollution with cognitive development in children. J. Epidemiol. Comm. H.

[60] Sugamata M., Ihara T., Umezawa M., Takeda K. (2012). P-999-Maternal exposure to nanoparticles enhances the risk of mental neurological disorders in offspring. Eur. Psychiat.

[61] Gloire G., Legrand-Poels S., Piette J. (2006). NF-κB activation by reactive oxygen species: Fifteen years later. Biochem. Pharmacol.

[62] Jomova K., Jenisova Z., Feszterova M., Baros S., Liska J., Hudecova D., Rhodes C., Valko M. (2011). Arsenic: Toxicity, oxidative stress and human disease. J. Appl. Toxicol.

[63] Peden D.B. (2011). The role of oxidative stress and innate immunity in O_3_ and endotoxin-induced human allergic airway disease. Immunol. Rev.

[64] Halliwell B., Gutteridge J.M.C. (1999). Free Radicals in Biology and Medicine.

[65] Schoonbroodt S., Piette J. (2000). Oxidative stress interference with the nuclear factor-kappa B activation pathways. Biochem. Pharmacol.

[66] Wang X., Martindale J.L., Liu Y., Holbrook N.J. (1998). The cellular response to oxidative stress: Influences of mitogen-activated protein kinase signalling pathways on cell survival. Biochem. J.

[67] Ornoy A. (2007). Embryonic oxidative stress as a mechanism of teratogenesis with special emphasis on diabetic embryopathy. Reprod. Toxicol.

[68] Wells P.G., Winn L.M. (1996). Biochemical toxicology of chemical teratogenesis. Crit. Rev. Biochem. Mol.

[69] Wells P.G., Bhuller Y., Chen C.S., Jeng W., Kasapinovic S., Kennedy J.C., Kim P.M., Laposa R.R., McCallum G.P., Nicol C.J. (2005). Molecular and biochemical mechanisms in teratogenesis involving reactive oxygen species. Toxicol. Appl. Pharm.

[70] Li Y., Liu Y., Fu Y., Wei T., Le Guyader L., Gao G., Liu R.S., Chang Y.Z., Chen C. (2011). The triggering of apoptosis in macrophages by pristine graphene through the MAPK and TGF-beta signaling pathways. Biomaterials.

[71] Meyer U., Feldon J., Fatemi S.H. (2009). *In-vivo* rodent models for the experimental investigation of prenatal immune activation effects in neurodevelopmental brain disorders. Neurosci. Biobehav. Rev.

[72] Tkachenko A.G., Xie H., Coleman D., Glomm W., Ryan J., Anderson M.F., Franzen S., Feldheim D.L. (2003). Multifunctional gold nanoparticle-peptide complexes for nuclear targeting. J. Am. Chem. Soc.

[73] Zhu L., Chang D.W., Dai L., Hong Y. (2007). DNA damage induced by multiwalled carbon nanotubes in mouse embryonic stem cells. Nano Lett.

[74] Li P.-W., Kuo T.-H., Chang J.-H., Yeh J.-M., Chan W.-H. (2010). Induction of cytotoxicity and apoptosis in mouse blastocysts by silver nanoparticles. Toxicol. Lett.

[75] Chan W., Shiao N. (2008). Cytotoxic effect of CdSe quantum dots on mouse embryonic development. Acta Pharmacol. Sin.

[76] Stelzer R., Hutz R.J. (2009). Gold nanoparticles enter rat ovarian granulosa cells and subcellular organelles, and alter *in vitro* estrogen accumulation. J. Reprod. Develop.

[77] Amatruda J.F., Shepard J.L., Stern H.M., Zon L.I. (2002). Zebrafish as a cancer model system. Cancer Cell.

[78] Hill A.J., Teraoka H., Heideman W., Peterson R.E. (2005). Zebrafish as a model vertebrate for investigating chemical toxicity. Toxicol. Sci.

[79] Kimmel C.B., Ballard W.W., Kimmel S.R., Ullmann B., Schilling T.F. (1995). Stages of embryonic development of the zebrafish. Dev. Dynam.

[80] Fadool J.M., Dowling J.E. (2008). Zebrafish: A model system for the study of eye genetics. Prog. Retin. Eye Res.

[81] Zon L.I., Peterson R.T. (2005). *In vivo* drug discovery in the zebrafish. Nat. Rev. Drug Discov.

[82] Selman K., Wallace R.A., Sarka A., Qi X. (1993). Stages of oocyte development in the zebrafish, Brachydanio rerio. J. Morphol.

[83] Bonsignorio D., Perego L., Giacco L.D., Cotelli F. (1996). Structure and macromolecular composition of the zebrafish egg chorion. Zygote.

[84] Lee K.J., Nallathamby P.D., Browning L.M., Osgood C.J., Xu X.H.N. (2007). *In vivo* imaging of transport and biocompatibility of single silver nanoparticles in early development of zebrafish embryos. ACS Nano.

[85] Nallathamby P.D., Lee K.J., Xu X.H.N. (2008). Design of stable and uniform single nanoparticle photonics for *in vivo* dynamics imaging of nanoenvironments of zebrafish embryonic fluids. ACS Nano.

[86] Cheng J., Flahaut E., Cheng S.H. (2007). Effect of carbon nanotubes on developing zebrafish (Danio rerio) embryos. Environ. Toxicol. Chem.

[87] Fent K., Weisbrod C.J., Wirth-Heller A., Pieles U. (2010). Assessment of uptake and toxicity of fluorescent silica nanoparticles in zebrafish (Danio rerio) early life stages. Aquat. Toxicol.

[88] Asharani P.V., lianwu Y., Gong Z., Valiyaveettil S. (2011). Comparison of the toxicity of silver, gold and platinum nanoparticles in developing zebrafish embryos. Nanotoxicology.

[89] George S., Lin S., Ji Z., Thomas C.R., Li L.J., Mecklenburg M., Meng H., Wang X., Zhang H., Xia T. (2012). Surface defects on plate-shaped silver nanoparticles contribute to its hazard potential in a fish gill cell line and zebrafish embryos. ACS Nano.

[90] Truong L., Tilton S.C., Zaikova T., Richman E., Waters K.M., Hutchison J.E., Tanguay R.L. (2013). Surface functionalities of gold nanoparticles impact embryonic gene expression responses. Nanotoxicology.

[91] Bar-Ilan O., Albrecht R.M., Fako V.E., Furgeson D.Y. (2009). Toxicity assessments of multisized gold and silver nanoparticles in zebrafish embryos. Small.

[92] Browning L.M., Lee K.J., Huang T., Nallathamby P.D., Lowman J.E., Xu X.H.N. (2009). Random walk of single gold nanoparticles in zebrafish embryos leading to stochastic toxic effects on embryonic developments. Nanoscale.

[93] Jovanović B., Ji T., Palić D. (2011). Gene expression of zebrafish embryos exposed to titanium dioxide nanoparticles and hydroxylated fullerenes. Ecotoxicol. Environ. Saf.

[94] Zhu X., Zhu L., Duan Z., Qi R., Li Y., Lang Y. (2008). Comparative toxicity of several metal oxide nanoparticle aqueous suspensions to Zebrafish (Danio rerio) early developmental stage. J. Environ. Sci. Health A.

[95] Bar-Ilan O., Louis K.M., Yang S.P., Pedersen J.A., Hamers R.J., Peterson R.E., Heideman W. (2012). Titanium dioxide nanoparticles produce phototoxicity in the developing zebrafish. Nanotoxicology.

[96] Truong L., Moody I.S., Stankus D.P., Nason J.A., Lonergan M.C., Tanguay R.L. (2010). Differential stability of lead sulfide nanoparticles influences biological responses in embryonic zebrafish. Arch. Toxicol.

[97] Lei Y., Xiao Q., Huang S., Xu W., Zhang Z., He Z., Liu Y., Deng F. (2011). Impact of CdSe/ZnS quantum dots on the development of zebrafish embryos. J. Nanopart. Res.

[98] Zhang W., Sun X., Chen L., Lin K.F., Dong Q.X., Huang C.J., Fu R.B., Zhu J. (2012). Toxicological effect of joint cadmium selenium quantum dots and copper ion exposure on zebrafish. Environ. Toxicol. Chem.

[99] Zhang W., Lin K., Miao Y., Dong Q., Huang C., Wang H., Guo M., Cui X. (2012). Toxicity assessment of zebrafish following exposure to CdTe QDs. J. Hazard. Mater..

[100] Zhang W., Lin K., Sun X., Dong Q., Huang C., Wang H., Guo M., Cui X. (2012). Toxicological effect of MPA–CdSe QDs exposure on zebrafish embryo and larvae. Chemosphere.

[101] King-Heiden T.C., Wiecinski P.N., Mangham A.N., Metz K.M., Nesbit D., Pedersen J.A., Hamers R.J., Heideman W., Peterson R.E. (2009). Quantum dot nanotoxicity assessment using the zebrafish embryo. Environ. Sci. Technol.

[102] Asharani P., Serina N., Nurmawati M., Wu Y., Gong Z., Valiyaveettil S. (2008). Impact of multi-walled carbon nanotubes on aquatic species. J. Nanosci. Nanotechnol.

[103] Cheng J., Cheng S.H. (2012). Influence of carbon nanotube length on toxicity to zebrafish embryos. Int. J. Nanomed.

[104] Usenko C.Y., Harper S.L., Tanguay R.L. (2007). *In vivo* evaluation of carbon fullerene toxicity using embryonic zebrafish. Carbon.

[105] Yamakoshi Y., Umezawa N., Ryu A., Arakane K., Miyata N., Goda Y., Masumizu T., Nagano T. (2003). Active oxygen species generated from photoexcited fullerene (C_60_) as potential medicines: O_2_
^−^.versus ^1^O_2_. J. Am. Chem. Soc.

[106] Usenko C.Y., Harper S.L., Tanguay R.L. (2008). Fullerene C_60_ exposure elicits an oxidative stress response in embryonic zebrafish. Toxicol. Appl. Pharm.

[107] Zhu R.R., Wang S.L., Chao J., Shi D.L., Zhang R., Sun X.Y., Yao S.D. (2009). Bio-effects of Nano-TiO_2_ on DNA and cellular ultrastructure with different polymorph and size. Mater. Sci. Eng. C.

[108] Gorth D.J., Rand D.M., Webster T.J. (2011). Silver nanoparticle toxicity in Drosophila: Size does matter. Int. J. Nanomed.

[109] Ispas C., Andreescu D., Patel A., Goia D.V., Andreescu S., Wallace K.N. (2009). Toxicity and developmental defects of different sizes and shape nickel nanoparticles in zebrafish. Environ. Sci. Technol.

[110] Carter A. (2007). Animal models of human placentation–a review. Placenta.

[111] Malassine A., Frendo J.L., Evain-Brion D. (2003). A comparison of placental development and endocrine functions between the human and mouse model. Hum. Reprod. Update.

